# Clinical and Demographic Characteristics of Patients Subjected to Physical Restraint in a Psychiatric Intensive Care Unit: A Retrospective Descriptive Cross-Sectional Study

**DOI:** 10.5152/eurasianjmed.2026.25961

**Published:** 2026-05-12

**Authors:** Cengiz Cengisiz, Sevgi Nehir

**Affiliations:** 1 Department of Psychiatry, Manisa Mental Health and Diseases Hospital, Manisa, Türkiye; 2 Department of Psychiatry Nursing, Manisa Celal Bayar University Faculty of Health Science, Manisa, Türkiye

**Keywords:** Agitation management, coercive measures, mental health, patient safety, physical restraint, psychiatric intensive care

## Abstract

**Background:**

Psychiatric intensive care units have significant implications for patient safety and clinical outcomes. Understanding the demographic and clinical characteristics of restrained patients is essential for developing evidence-based protocols and reducing unnecessary restraint use. The aim of this retrospective descriptive cross-sectional study was to determine the clinical and demographic characteristics of patients subjected to physical restraint in a psychiatric intensive care unit.

**Methods:**

This descriptive cross-sectional study analyzed 138 patients subjected to physical restraint in a psychiatric intensive care unit between January 2023 and December 2023. Data were collected through systematic chart review using a standardized physical restraint monitoring form from the Hospital Quality and Standards System, designed specifically for type 2 (behavioral safety) restraint documentation. Variables included demographic characteristics, primary diagnoses, restraint indications, and clinical outcomes. Statistical analysis was performed using SPSS, version 28.0. The study followed STROBE (Strengthening the Reporting of Observational Studies in Epidemiology) guidelines. Statistical analysis was performed using descriptive statistics.

**Results:**

Out of 138 patients, 95 (68.8%) were male with a mean age of 51.48 ± 15.02 years. Primary restraint indications included noncompliance with medical treatment (39 patients, 28.3%), severe agitation (37 patients, 26.8%), orientation disturbances (32 patients, 23.2%), and self-harm behaviors (30 patients, 21.7%). The most common primary diagnoses were nonorganic psychosis (55 patients, 39.9%), bipolar disorder (30 patients, 21.7%), and delirium tremens (20 patients, 14.5%). Mean intensive care unit length of stay was 11.87 ± 14.04 days.

**Conclusion:**

This study provides essential baseline data on physical restraint use in psychiatric intensive care, demonstrating male predominance and identifying key clinical indicators. These findings support the development of targeted interventions and evidence-based restraint protocols to optimize patient care while minimizing restrictive practices.

**Main Points**

- Male patients comprised 68.8% of those subjected to physical restraint in psychiatric intensive care, with noncompliance with medical treatment being the most common indication (28.3%).
- Nonorganic psychosis was the predominant primary diagnosis (39.9%), followed by bipolar disorder (21.7%) and delirium tremens (14.5%).
- The mean intensive care unit length of stay was 11.87 ± 14.04 days, indicating the complex nature of cases requiring physical restraint.
- These findings provide essential baseline data for developing evidence-based restraint reduction strategies and targeted interventions in psychiatric intensive care settings.

## Introduction

Physical restraint in psychiatric settings represents one of the most challenging aspects of mental healthcare, balancing immediate safety concerns with patient rights and therapeutic relationships. Physical restraint is the temporary restriction of a patient’s movements using manual or mechanical means to mitigate the risk of harm to self or others. It should be considered within a least-restrictive framework and only after systematic de-escalation efforts have proven insufficient. Despite ongoing efforts to reduce restraint use globally, these interventions remain necessary in specific clinical situations where alternative de-escalation strategies have proven insufficient.^1^^,^^2^

Psychiatric intensive care units (PICUs) serve as specialized environments for patients requiring the highest level of psychiatric care, typically those presenting with severe behavioral disturbances, acute psychosis, or significant risk of self-harm or violence toward others. The concentrated nature of high-acuity patients in these units necessitates frequent consideration of restrictive interventions,^3^^,^^4^ making PICUs critical settings for understanding restraint utilization patterns.

International research has identified several demographic and clinical factors associated with increased restraint use, including male gender, younger age, psychotic disorders, substance use disorders, and previous psychiatric hospitalizations.^5^^-^^7^ Recent systematic evidence highlights substantial international variation in mechanical restraint practices, with prevalence estimates ranging from 1.4% to 58.7% across inpatient settings. A comprehensive systematic review by Whiting et al^8^ synthesized global data demonstrating that male gender, psychotic disorders, and greater symptom severity constitute consistent risk factors for restraint use, while also documenting significant heterogeneity in healthcare system approaches. Recent analyses also highlight the importance of addressing potential biases in violence risk assessments to avoid unnecessary stigmatization of psychiatric populations.^9^ Similarly, meta-analytic evidence from 77 studies^10^ reported pooled prevalence estimates of 14.4% for physical restraint, with marked variability influenced by cultural, legal, and institutional factors.

In Türkiye, restraint practices reflect specific systemic and clinical contexts. Emergency department data from a Turkish mental health hospital reported a 3.51% physical restraint prevalence among 2051 psychiatric presentations, with a median duration of 90 minutes and significant male predominance.^11^ Comparative studies further indicate that Turkish psychiatry wards rely more heavily on mechanical restraint compared to European services, where seclusion is often preferred as a containment strategy.^12^ Additionally, regional studies have documented frequent restraint use with substantial inter-ward variability, suggesting that restraint forms part of routine clinical practice rather than exceptional crisis intervention.^13^^,^^14^

Despite these contributions, data specifically characterizing patients subjected to physical restraint in Turkish psychiatric intensive care units remain scarce. Given that PICUs concentrate the highest-acuity patients with severe behavioral disturbances, understanding local demographic and clinical patterns is essential for developing context-specific, evidence-based protocols and aligning with international least-restrictive practice standards.^15^

However, significant variations exist across healthcare systems, cultural contexts, and institutional policies, emphasizing the need for local data to inform evidence-based practice.^16^^,^^17^

The clinical indications for physical restraint in psychiatric settings typically include imminent danger to self or others, severe agitation unresponsive to verbal intervention, and medical necessity when patients cannot cooperate with essential treatments.^18^^,^^19^ Understanding the specific patterns of these indications within local contexts is essential for developing targeted quality improvement initiatives and staff training programs.^20^^,^^21^

Despite the growing body of international literature on restraint use, limited data exist regarding the demographic and clinical characteristics of patients subjected to physical restraint in Turkish psychiatric intensive care settings. This knowledge gap represents a significant barrier to implementing evidence-based restraint reduction strategies and optimizing patient care protocols.

The objective of this descriptive cross-sectional study was to determine the clinical and demographic characteristics of patients subjected to physical restraint in a psychiatric intensive care unit, with the goal of providing essential baseline data to inform clinical practice improvements and quality assurance initiatives.

## Material and Methods

### Study Design and Setting

This study is retrospective cross-sectional research. The study was conducted in a 12-bed psychiatric intensive care unit within a tertiary care hospital. The study followed the Strengthening the Reporting of Observational Studies in Epidemiology (STROBE) guidelines for reporting observational studies.^22^ Data collection covered the period from January 1, 2023, to December 31, 2023, ensuring comprehensive capture of seasonal variations and clinical patterns.

### Participants

The study population comprised all patients (N = 138) who experienced at least 1 episode of physical restraint during their PICU admission within the study period. Inclusion criteria were (1) admission to the psychiatric intensive care unit; (2) age ≥18 years; (3) documented use of physical restraint; and (4) complete medical records available for analysis. Exclusion criteria included patients with incomplete documentation (n = 3) or those transferred before restraint evaluation could be completed (n = 2). This represents consecutive sampling of all eligible cases during the study period.

### Data Collection

Primary diagnoses were coded according to International Statistical Classification of Diseases and Related Health Problems, 10th Revision (ICD-10) criteria, as used in Turkish healthcare settings, then mapped to Diagnostic and Statistical Manual of Mental Disorders, Fifth Edition, Text Revision (DSM-5-TR) equivalents for international readability and comparison with global literature. Specific mapping was as follows: ICD-10 F29 (unspecified nonorganic psychosis) was mapped to DSM-5-TR 298.9 (unspecified schizophrenia spectrum and other psychotic disorder); ICD-10 F31 (bipolar affective disorder) was mapped to DSM-5-TR 296.80 (bipolar and related disorders); and ICD-10 F10.4 (withdrawal state with delirium) was mapped to DSM-5-TR 291.0 (alcohol withdrawal delirium). Discrepancies between the 2 psychiatrists were resolved through consensus.

This study examined type 2 (behavioral safety) physical restraints only. During the study period (2023), physical restraint in the PICU was applied exclusively for behavioral safety indications: severe agitation, violence/self-harm risk, and acute orientation disturbances. Type 1 (treatment-support) mechanical restraints were not utilized, as psychiatric intensive care in Türkiye focuses on behavioral stabilization rather than medical-surgical interventions requiring physical immobilization. Data extraction utilized the standardized physical restraint monitoring form mandated by the Hospital Quality and Standards System. This form is specifically designed for type 2 (behavioral safety) restraint episodes and captures: patient demographics, primary diagnosis (ICD-10 coded), documented indication for restraint, restraint initiation/termination times, and immediate outcomes. The form is completed by attending psychiatrists for all physical restraint episodes as part of routine quality assurance protocols.

Regarding chemical restraint: While pharmacological sedation (e.g., haloperidol, benzodiazepines) is frequently ordered for acute agitation and irritability in this tertiary regional psychiatric hospital setting, these agents were not recorded as a distinct restraint category in the electronic health system. Instead, they were documented as standard PRN (Pro re nata) medications within routine treatment protocols. Consequently, reliable differentiation between chemical restraint and maintenance therapy was not feasible, and this study focuses exclusively on physical (mechanical) restraint episodes.

### Statistical Analysis

This retrospective cross-sectional study analyzed quantitative data using SPSS, version 28.0 (IBM SPSS Corp.; Armonk, NY, USA). Continuous variables are presented as means ± SD, and categorical variables as frequencies and percentages. For restraint indications, when multiple reasons were documented, the clinically predominant indication, as determined by the attending psychiatrist, was coded as the primary reason.

### Ethical Considerations

This study was approved by the Health Sciences Ethics Committee of Manisa Celal Bayar University Faculty of Medicine (Approval number: 20.478.486/1611, Date: December 21, 2022) and conducted in accordance with the Declaration of Helsinki (as revised in 2013). Given the retrospective design utilizing de-identified archival records, the committee granted a waiver of individual informed consent in accordance with national research ethics guidelines and international ethical standards.

## Results

During the study period, 138 patients experienced physical restraint in the psychiatric intensive care unit. The demographic and clinical characteristics of these patients are presented in Table 1.

### Demographic Characteristics

The mean age of restrained patients was 51.48 ± 15.02 years (range: 20-87 years). Males comprised 68.8% (n = 95) of the study population, while females represented 31.2% (n = 43). Given the clinical significance of delirium tremens as a high-mortality condition in psychiatric intensive care, it was separately reported that the delirium tremens subgroup (n = 20, 14.5%) had a mean age of 51.80 ± 9.63 years (range: 38-71 years).

### Clinical Characteristics

The most prevalent primary diagnosis was nonorganic psychosis (39.9%, n = 55), followed by bipolar disorder (21.7%, n = 30) and delirium tremens (14.5%, n = 20). Schizophrenia accounted for 8.0% (n = 11) of cases, while personality disorders represented 4.3% (n = 6) of the sample.

### Restraint Indications

For patients subjected to physical restraints in the intensive care unit, noncompliance with medical treatment was the most common indication (n = 39, 28.3%). This was followed by severe agitation (n = 37, 26.8%), orientation disturbances (n = 32, 23.2%), and self-harm behaviors (n = 30, 21.7%) (total N = 138). These 4 categories accounted for the majority of restraint episodes in the psychiatric intensive care unit.

### Healthcare Utilization

Most patients (77.5%, n = 107) experienced a single intensive care unit (ICU) admission during the study period. Multiple admissions occurred in 22.5% of cases, with 17.4% (n = 24) having 2 admissions, 2.9% (n = 4) having 3 admissions, 1.4% (n = 2) having 4 admissions, and 0.7% (n = 1) having 5 admissions. The mean ICU length of stay was 11.87 ± 14.04 days (range: 1-88 days).

Judicial admission status was present in 8.0% (n = 11) of cases, while 92.0% (n = 127) were voluntary or family-initiated admissions.

## Discussion

The objective of this descriptive cross-sectional study was to determine the clinical and demographic characteristics of patients subjected to physical restraint in a psychiatric intensive care unit. The present findings demonstrate a clear male predominance (68.8%) among restrained patients, consistent with international literature reporting higher restraint rates in male psychiatric patients.^23^^,^^24^ These findings of male predominance (68.8%) align with international evidence identifying male gender as a consistent predictor of mechanical restraint across diverse inpatient settings.^9^^,^^25^ However, the mean age of 51.48 years in the current sample represents a notable deviation from international studies typically reporting younger populations (mean ages 30-40 years).^25^ This discrepancy likely reflects 2 specific features of the PICU population: first, the substantial representation of delirium tremens cases (14.5%), which typically occurs in middle-aged individuals with chronic alcohol dependence;^26^ second, potential differences in PICU admission criteria between healthcare systems, where the present unit may admit older patients with complex medical-psychiatric comorbidities. This elevated age profile may also reflect regional demographic patterns in alcohol-related disorders and the tertiary referral function of the center, which receives complex cases from surrounding provinces.^27^^,^^28^ This age distribution may reflect the specific demographic characteristics of the healthcare region or could indicate different patterns of psychiatric service utilization in the present setting.

The diagnostic distribution in this study reveals important clinical patterns. Nonorganic psychosis was the most common primary diagnosis (39.9%), followed by bipolar disorder (21.7%) and delirium tremens (14.5%). The substantial representation of delirium tremens cases (14.5%) highlights the dual role of the PICU in managing both primary psychiatric conditions and substance-related medical emergencies requiring intensive psychiatric intervention.^29^^,^^30^ The diagnostic distribution, with nonorganic psychosis (39.9%) and bipolar disorder (21.7%) as leading categories, mirrors single-site European studies reporting these diagnoses as primary correlates of mechanical restraint.^31^ Nevertheless, the high proportion of delirium tremens (14.5%) distinguishes the present sample from general psychiatric inpatient populations and highlights the dual medical-psychiatric function of PICUs in managing substance-related emergencies.^26^ The present analysis of restraint indications provides crucial insights for clinical practice improvement. Noncompliance with medical treatment emerged as the leading indication (28.3%), followed closely by severe agitation (26.8%). This pattern suggests that a significant proportion of restraint use is driven by the need to ensure medical treatment completion rather than solely for behavioral control, indicating opportunities for enhanced therapeutic engagement strategies.

Compared with Turkish emergency department data reporting 3.51% restraint prevalence with agitation as the primary indication and a median duration of 90 minutes,^11^ the PICU sample showed a different clinical profile: higher rates of noncompliance with medical treatment (28.3%) and orientation disturbances (23.2%), with a substantially longer mean length of stay. This shift in indication patterns suggests that PICU-restrained patients represent a more complex, medically compromised subgroup requiring distinct intervention protocols beyond acute behavioral control. These findings align with international observations that intensive care units manage higher-acuity populations with greater medical complexity than general emergency or acute ward settings.^9^

Furthermore, to properly contextualize the PICU findings within the broader national landscape, key Turkish studies examining restraint in general psychiatric settings were referenced. Sercan^13^ documented frequent mechanical restraint use with substantial inter-ward variability in a regional mental health hospital, establishing that restraint forms part of routine clinical practice rather than exceptional crisis intervention in Turkish general adult psychiatry. Similarly, Coşkun and Avlamaz^14^ reported significant utilization of mechanical restraints with heterogeneous restraining periods across acute psychiatric clinics in a 1-year period. However, the PICU data diverge critically from these general ward profiles: while those studies described restraint primarily for acute behavioral control in standard settings, the intensive care unit demonstrated a distinct clinical profile with substantially higher rates of noncompliance with medical treatment (28.3%) and orientation disturbances (23.2%). This suggests that PICU-restrained patients represent a more medically complex, high-acuity subgroup requiring specialized intervention protocols distinct from those applicable to general psychiatric wards or emergency departments.

The relatively high frequency of orientation disturbances (23.2%) as a restraint indication aligns with the substantial delirium tremens population and may reflect the cognitive impairment associated with acute substance withdrawal states. This prevalence underscores the necessity of continuous observation protocols and structured risk assessment tools to mitigate self-injury risk in high-acuity psychiatric settings.^32^^,^^33^Self-harm behaviors accounted for 21.7% of restraint indications, emphasizing the critical safety considerations inherent in PICU practice.^32^^,^^33^

The mean ICU length of stay (11.87 ± 14.04 days) demonstrates the intensive nature of care required for this population. The substantial SD indicates considerable variability in treatment duration, likely reflecting the heterogeneity of clinical presentations and treatment responses among restrained patients.^34^^,^^35^ The wide range in ICU length of stay (1-88 days) and association with restraint use is consistent with recent work demonstrating that greater clinical severity, comorbidity, and exposure to coercive measures predict prolonged hospitalization in psychiatric units.^36^ This variability likely reflects the heterogeneous nature of treatment-resistant presentations and complex substance-related emergencies requiring extended stabilization periods.

The low rate of judicial admissions (8.0%) suggests that most restrained patients were admitted through voluntary or family-initiated pathways, indicating that restraint use was not primarily associated with involuntary commitment status in the present setting.^8^^,^^37^^-^^42^

Consistent with evidence-based best-practice guidelines emphasizing mechanical restraint as a last resort following systematic de-escalation attempts,^15^ and supported by empirical data demonstrating that coercive measures are associated with significantly poorer mental health status at discharge—as evidenced by increased symptom severity on the Health of the Nation Outcome Scales^43^—these findings suggest that minimizing restrictive interventions remains essential given the potential for adverse clinical outcomes. The high proportion of noncompliance indications (28.3%) specifically indicates opportunities for enhanced therapeutic engagement strategies to reduce reliance on physical containment.

### Strengths and Clinical Implications

This study has several notable strengths. It provides the first systematic characterization of restrained patients in Turkish psychiatric intensive care, filling a critical evidence gap in an understudied setting. The census sampling approach minimizes selection bias, and the 1-year study duration captures seasonal variations in clinical presentations. The dual ICD-10/DSM-5-TR diagnostic mapping enhances international comparability, while the explicit focus on type 2 behavioral safety restraints clarifies the clinical scope.

These findings have several important implications for clinical practice. First, the identification of noncompliance with medical treatment as the primary restraint indication suggests that enhanced patient engagement strategies, therapeutic communication techniques, and motivational interviewing approaches may be valuable for restraint reduction. Second, the male predominance indicates the need for gender-specific de-escalation training and environmental modifications.

The substantial representation of delirium tremens cases suggests the need for specialized protocols addressing substance withdrawal management in the PICU setting, including pharmacological strategies that may reduce behavioral complications requiring restraint.

### Limitations and Future Directions

Several limitations should be acknowledged. First, this was a single-center study, which may limit the generalizability of findings to other psychiatric intensive care settings. Second, the retrospective design relies on documentation quality and may be subject to information bias. Third, restraint duration, frequency of episodes per patient, or outcomes following restraint use were not analyzed; these would have provided additional insights into restraint patterns and effectiveness.

Additionally, a comparison group of non-restrained PICU patients was not included, which would have enabled identification of specific risk factors for restraint use. Cross-coding of restraint indications by diagnostic categories was not performed, precluding indication-by-diagnosis breakdowns. Future prospective studies should implement standardized coding schemas to enable comprehensive risk stratification by clinical subgroups. Future studies should employ prospective designs with standardized data collection instruments and include comparison groups to enhance the evidence base for restraint reduction strategies.

This study provides essential baseline data for an understudied setting—Turkish psychiatric intensive care—where no prior characterization of restrained patients existed. Future research should build upon these baseline findings through prospective multicenter designs with standardized instruments. Specifically, implementation studies evaluating the 4 evidence-based intervention categories identified by Pedersen et al^44^—calm-down methods, enhanced staff resources, policy changes, and culture-change programs—would translate descriptive data into clinical protocols. Additionally, developing a standardized taxonomy for restraint subtypes and chemical sedation in Turkish electronic health systems (MEDULA [The National Health Service electronic billing and automation system in Türkiye]) would enable comparative outcome analyses. Studies targeting the presently identified high-risk subgroups (delirium tremens patients and those with treatment noncompliance) through symptom-triggered withdrawal protocols and shared decision-making interventions are particularly warranted.

## Conclusion

This descriptive cross-sectional study aimed to characterize the clinical and demographic profile of psychiatric intensive care patients subjected to physical restraint. The present analysis successfully characterized this specialized population and identified primary restraint indications, providing essential local context for evidence-based practice implementation.

This study successfully characterized the clinical and demographic profile of psychiatric intensive care patients subjected to physical restraint, demonstrating male predominance and identifying noncompliance with medical treatment as the leading indication. These findings provide essential baseline data for developing context-specific, evidence-based restraint reduction strategies in Turkish psychiatric intensive care settings.

## Figures and Tables

**Table 1.** Demographic and Clinical Characteristics of Individuals (N = 138)

**Characteristic**	**n**	**%**
Age group (mean 51.48 ± 15.02; min 20, max 87)		
51 years and younger	59	42.8
52 years and older	79	57.2
Delirium tremens subgroup age (51.80 ± 9.63; min 38, max 71)	20	14.5
Gender		
Female	43	31.2
Male	95	68.8
Primary diagnosis		
Nonorganic psychosis	55	39.9
Schizophrenia	11	8.0
Bipolar disorder	30	21.7
Nonorganic psychosis with intellectual disability	1	0.7
Personality disorders	6	4.3
Delirium tremens	20	14.5
Substance use disorder	5	3.6
Acute psychotic disorder	6	4.3
Anxiety disorder	3	2.2
Conversion disorder	1	0.7
Restraint indications		
Noncompliance with medical treatment	39	28.3
Severe agitation	37	26.8
Orientation disturbances	32	23.2
Self-harm behaviors	30	21.7
Judicial admission		
Available	11	8.0
None	127	92.0
Number of ICU admissions		
1	107	77.5
2	24	17.4
3	4	2.9
4	2	1.4
5	1	0.7
ICU length of stay (days): 11.87 ± 14.04 (min 1, max 88)		

For each case, a primary indication was coded; when multiple reasons were present, the clinically predominant reason was reported. Diagnostic mapping: ICD-10 diagnoses mapped to DSM-5-TR equivalents for international readability.

Classification footnote: All 138 episodes represent type 2 (behavioral safety) physical restraints applied for behavioral control. Type 1 (treatment-support) restraints were not utilized in this setting.

ICU, intensive care unit.

## Data Availability

The data that support the findings of this study are available on request from the corresponding author.
